# Aortic valve replacement via a right parasternal approach in a patient with a history of coronary artery bypass surgery and pericardiectomy: a case report

**DOI:** 10.1186/s40792-019-0598-5

**Published:** 2019-03-04

**Authors:** Takuya Nakayama, Miki Asano

**Affiliations:** Department of Cardiovascular Surgery, Nagoya Kyoritsu Hospital, 1-172 Hokke, Nakagawa, Nagoya, 454-0933 Japan

**Keywords:** Minimally invasive, Aortic valve replacement, Coronary artery bypass surgery, Right parasternal approach

## Abstract

**Background:**

The number of patients who require aortic valve replacement after coronary artery bypass grafting continues to increase. Re-operative cardiovascular surgery after coronary artery bypass grafting has various risk factors related to median re-sternotomy. It is particularly essential to avoid damage to the living graft. We successfully performed aortic valve replacement via right parasternal thoracotomy in a patient who had undergone coronary artery bypass grafting.

**Case presentation:**

An 80-year-old man who had undergone coronary artery bypass grafting was referred to our hospital for syncope caused by severe aortic valve stenosis. He also had a history of pericardiotomy for constrictive pericarditis. His left internal thoracic artery bypass graft was patent. Aortic valve replacement was performed through a small right parasternal thoracotomy during cardiac arrest following cardiopulmonary bypass under moderate hypothermia and hyperkalemia by intermittent selective antegrade cardioplegia. His postoperative course was uneventful.

**Conclusion:**

Aortic valve replacement via right parasternal thoracotomy with moderate hypothermia and hyperkalemia was safe and effective for avoidance of re-sternotomy-related complications.

## Background

The number of patients who require aortic valve replacement for aortic valve stenosis (AS) long after coronary artery bypass grafting (CABG) continues to increase [1]. Re-operative cardiovascular surgery after CABG is more complicated and hazardous than the initial surgery because of risk factors related to median re-sternotomy, such as damage to the heart and/or grafts associated with division of sternal adhesions [2, 3]. Transcatheter aortic valve replacement (TAVR) is now a widely accepted therapeutic modality and is another option for this subset of patients. Hospital mortality associated with TAVR is similar to that associated with open heart aortic valve replacement (AVR) in patients who have previously undergone CABG. However, the rates of permanent pacemaker implantation and paravalvular leakage are higher in patients undergoing TAVR [4, 5]. Therefore, AVR after CABG might have fewer risks if re-sternotomy can be avoided. We performed minimally invasive AVR via right parasternal thoracotomy in a patient with history of CABG.

## Case presentation

An 80-year-old man was referred to our hospital for syncope caused by severe AS. Twelve years previously, he had undergone CABG that comprised bypass grafting of the left internal thoracic artery (LITA) to the left anterior descending coronary artery (LAD) and of the saphenous vein from the ascending aorta to circumflex branch. He had also undergone pericardiectomy for constrictive pericarditis 10 years prior to the surgery. Unfortunately, the details of the surgical procedure and findings were unknown because the surgery for pericarditis was performed at another hospital. Preoperative computed tomography indicated that the pericardium around the aorta and right-sided left atrial area were almost intact. However, severe adhesion appeared to be present from the anterior to diaphragmatic aspects of the heart. Echocardiography showed severe progressive AS with moderate aortic regurgitation. Other examination data were as follows: aortic valve area of 0.6 cm^2^, mean trans-aortic valvular pressure gradient of 86 mmHg, bicuspid aortic valve, and left ventricular ejection fraction of 70%. Although the patency of the LITA–LAD graft was confirmed, computed tomography and coronary arteriography showed that the saphenous vein graft was occluded. We discussed the treatment strategy (TAVR or AVR) in a “heart team.” The heart team considered TAVR not to be suitable for his deformed bicuspid aortic valve. We decided to use a right parasternal minimally invasive approach, which is optimal for performing AVR to avoid median sternotomy-related injury, especially to the patent LITA–LAD graft.

**Fig. 1:**
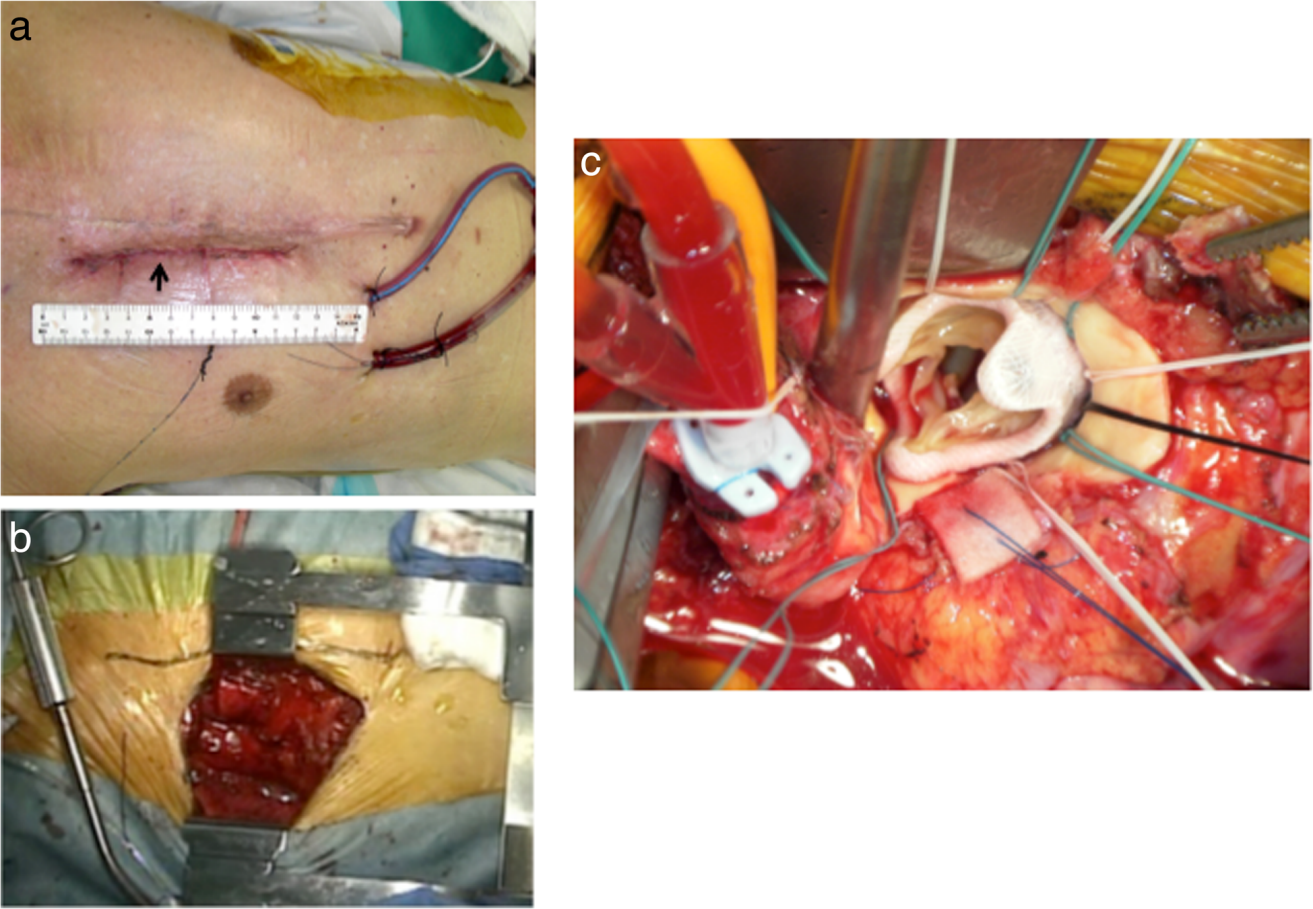
**a** An approximately 10-cm right parasternal incision was made (arrow). **b** Next, pericardial stay sutures were placed, providing an excellent view of the ascending aorta. **c** A pericardial bioprosthesis was implanted. The view of the operative field was excellent

A 7-cm right parasternal incision extending from the inferior edge of the second costal cartilage to the superior edge of the fourth costal cartilage was made (Fig. 1a). Both the third and fourth costal cartilages were totally excised following exposure of the second and third intercostal spaces by division of the pectoralis major muscle. The right ITA was ligated immediately inferior to the second costal cartilage and immediately superior to the fifth costal cartilage. The intercostal muscles and pleura were incised. The pericardium around the aorta was intact as estimated by computed tomography, and the adhesion around the aorta was less severe than predicted preoperatively. Pericardial stay sutures were placed, providing excellent exposure of the ascending aorta. Next, the ascending aorta was exposed and controlled (Fig. 1b). Cardiopulmonary bypass (CPB) using the femoral artery and vein was initiated. A left ventricular vent cannula was placed in the right superior pulmonary vein, and then the patient was cooled to 28 °C. Because of severe adhesion around the right atrium, a retrograde catheter could not be inserted. We only injected antegrade cardioplegia solution after the ascending aorta was cross-clamped. Once cardioplegic arrest was obtained, ventricular fibrillation developed. Therefore, we administered 40 meq/L potassium via the CPB to maintain a blood potassium concentration of 8 meq/L. Antegrade cold blood cardioplegia was induced intermittently every 20 min. A 19-mm Mosaic pericardial bioprosthesis (Medtronic, Minneapolis, MN, USA) was implanted (Fig. 1c). After the patient had been placed in the Trendelenburg position, the aorta was unclamped and de-airing was accomplished through suction on the cardioplegia aortic root needle with flooding of CO_2_ gas in the operative field. The aortic cross-clamping time was 83 min. A ventricular pacemaker wire was placed in the right ventricle while the CPB was running, and the heart was decompressed. The patient was smoothly separated from CPB. The operation time and CPB time were 348 and 158 min, respectively. Immediately after surgery, the absence of ischemic damage to the myocardium was confirmed based on the serum creatine kinase MB concentration and electrocardiography findings. Echocardiography also showed normal movement of the left ventricle. The postoperative course was uneventful, and the patient was discharged on postoperative day 7.

## Discussion

The number of patients with a history of CABG who require aortic valve surgery has recently been increasing [1]. Redo cardiovascular surgery after CABG surgery is more complicated than the initial surgery and may also be more hazardous because of risk factors related to median re-sternotomy, such as cardiac injury and damage to patent grafts associated with division of sternal adhesions. The operative mortality rate associated with AVR after CABG reportedly ranges from 6 to 16%. Dissection and clamping of ITA grafts is generally performed for sufficient myocardial protection during AVR. However, this is associated with graft injury in 5 to 50% of cases and thus a poor prognosis [2, 3]. Tabata et al. reported that myocardial protection during cardiac arrest in patients with patent LITA grafts could be accomplished by systemic hypothermia and hyperkalemia as well as implementation of bilateral (antegrade and retrograde) cardioplegia without clamping of living grafts [1, 6]. In our case, cardiac arrest was obtained with only antegrade cardioplegia under mild hypothermia at a minimal temperature of 28 °C. It was not difficult to accomplish and maintain cardiac arrest to perform all scheduled procedures.

Various approaches except median sternotomy have been used for AVR recently. Cosgrove et al. described the procedure involved in performing the right parasternal approach [7, 8]. In our institution, a right parasternal approach is the first choice for minimally invasive AVR because of its versatility and safety without sternotomy. We also performed double-valve replacement, replacement of the ascending aorta, and CABG for the right coronary artery with this approach. A right parasternal approach is thought to be suitable for redo cardiac surgery after CABG because surgical synechiotomy is minimized without involving an ITA graft.

TAVR might be a very good option in patients with a history of CABG who require AVR. Although the incidence of paravalvular leakage, a significant complication of TAVR, has recently decreased, various complications are still possible [4, 5]. We suggest that a right parasternal approach without re-sternotomy is an excellent and safe alternative surgical approach for AVR in patients who have undergone CABG, especially at facilities that do not perform TAVR as effectively as us. Of course, this treatment modality should involve moderate hypothermia and hyperkalemia in association with cardiac arrest by intermittent bilateral or selective antegrade cardioplegia.

## Conclusion

Avoidance of re-sternotomy, the lack of contact with the ITA graft, the use of moderate hypothermia and hypothermia, and the use of intermittent selective antegrade cardioplegia make this procedure an optimal treatment option for patients who require aortic valve replacement after coronary artery bypass grafting.

## Abbreviations

AS: Aortic valve stenosis
AVR: Aortic valve replacement
CABG: Coronary artery bypass grafting
CPB: Cardiopulmonary bypass
LAD: Left anterior descending coronary artery
LITA: Left internal thoracic artery
TAVR: Transcatheter aortic valve replacement
